# Liquid-crystalline heterodimesogens and ABA-heterotrimesogens comprising a bent 3,5-diphenyl-1,2,4-oxadiazole central unit

**DOI:** 10.3762/bjoc.8.54

**Published:** 2012-03-30

**Authors:** Govindaswamy Shanker, Marko Prehm, Carsten Tschierske

**Affiliations:** 1 Institute of Chemistry, Organic Chemistry, Martin-Luther-University Halle-Wittenberg, Kurt Mothes Str. 2, D-06120 Halle/Saale, Germany, Tel: ++49 (0) 345 55 25664, Fax: ++49 (0) 345 55 27346

**Keywords:** bent-core mesogens, cybotactic nematic phases, dimesogen, liquid crystals, 1,2,4-oxadiazoles, trimesogen

## Abstract

Three new types of terminally connected ABA-heterotrimesogens and heterodimesogens, composed of a bent 3,5-diphenyl-1,2,4-oxadiazole central unit and one or two rod-shaped 4-cyanobiphenyl cores or one 2-phenyl-1,3,4-thiadiazole core, connected by flexible spacers, have been synthesized, and their mesomorphic behavior was studied by optical polarizing microscopy (PM), differential scanning calorimetry (DSC) and X-ray diffraction (XRD). All dimesogens exhibit broad ranges of cybotactic nematic phases (N_cybA_ and N_cybC_), in some cases accompanied by additional mesophases (CybA or SmC) at lower temperature. The combination of the 3,5-diphenyl-1,2,4-oxadiazole unit with one cyanobiphenyl core leads to the removal of tilted smectic and cybotactic nematic phases (SmC, N_cybC_), which are replaced by the nontilted CybA phases and nematic phases composed of SmA-type clusters (N_cybA_). The orthogonal cybotactic nematic phases of bent-core mesogens are of special interest for achieving biaxial nematic phases of the orthorhombic type. The orthogonal (N_cybA_) and skewed (N_cybC_) cybotactic nematic phases were distinguished by XRD and optical observations.

## Introduction

Liquid-crystalline (LC) dimers of low-molar-mass compounds formed by the coupling of two mesogenic segments are of contemporary interest [1–4]. These compounds exhibit fascinating properties, often different from the single mesogens, and they can also be considered as model compounds for main-chain LC polymers [5–8]. Dimesogens can be categorized into mesogenic dimers (homodimesogens), composed of identical mesogenic units, and heterodimesogens combining different types of units, such as two distinct rod-like units [4], or combining rod-like with discotic [1,9–10], phasmidic or bent-core units, linked together through flexible chains [1]. These flexible chains, which in most cases represent linear alkyl chains, connect the distinct mesogenic units, but depending on their length and degree of flexibility they can also decouple the segmental motions of the two interconnected mesogenic units, and hence these chains are also assigned as spacers. The properties of these dimesogens strongly depend on the topology of connection [11], the spacer length and spacer parity, resulting in linear and bent [1,3], T-shaped [12–14], Y-shaped [15] or H-shaped molecules [12–13,16–19]. Moreover, combinations of mesogenic units differing not only in shape [20–22], but also in polarity or compatibility of the mesogenic units [1–4,23], and dimesogens incorporating chiral [24–26] segments often result in unique interesting properties, which are fundamentally different from the individual mesogens.

Recent interest in dimesogens has been focused on combinations of two rod-like molecules through relatively short odd-numbered spacer units to obtain dimesogens with an overall bent shape [27–31] exhibiting properties that are characteristic for bent-core mesogens, such as polar ferroelectric or antiferroelectric switching LC phases [32–35] and spontaneous achiral symmetry breaking [34–36]. Also dimesogens combining two bent-core units terminally [37–43] or laterally [44], or combining a bent-core segment with a rod-shaped mesogen [40,45–54], and related end-to-end connected ABA-heterotrimesogens combining a bent-core segment with two rod-like units [55], or a rod-like core with two bent-core units [56], were synthesized and studied. These compounds are of interest with respect to the potential biaxiality of the nematic (N) [45,50,57] and nontilted smectic phases of these compounds [45]. Bent-core units have also been combined with disc-like units, but in this case the incompatibility and steric mismatch of the distinct units led to a loss of LC properties [58].

The 3,5-diphenyl-1,2,4-oxadiazole segment has recently attracted significant attention as a central building block for bent-core LC molecules (angle ~140°) [59–63], due to the ferroelectric-like polar switching observed in the nematic phases of some of these compounds under applied electric fields [64–65]. However, these nematic phases were only observed at high temperature, which makes their investigation difficult.

**Scheme 1:**
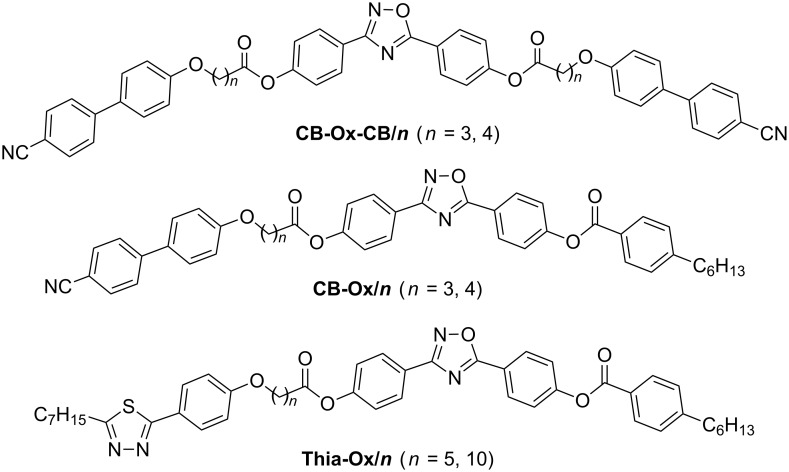
Structures of the investigated ABA-heterotrimesogens **CB-Ox-CB/*n*** and heterodimesogens **CB-Ox/*n***, **Thia-Ox/*n***.

**Scheme 2:**
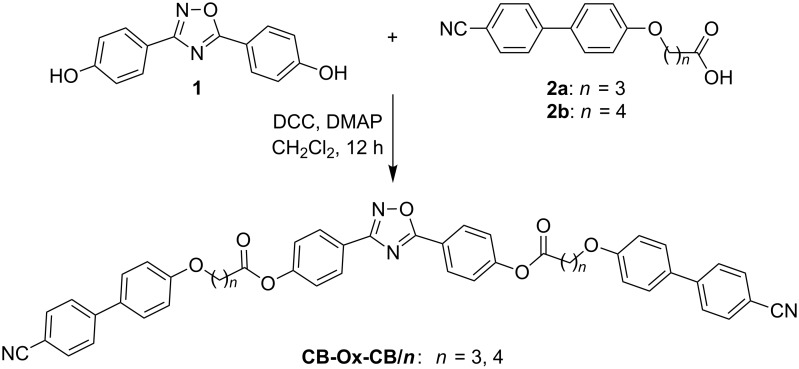
Synthesis of the ABA-heterotrimesogens **CB-Ox-CB/*n***.

**Scheme 3:**
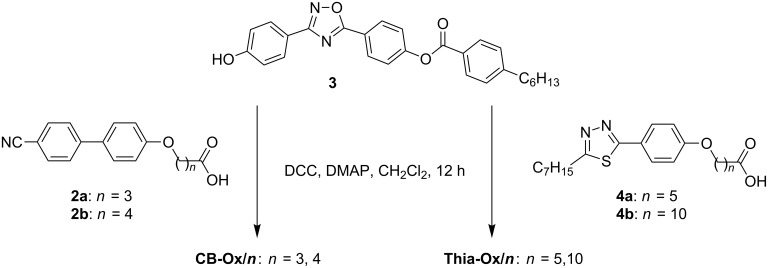
Synthesis of the dimesogens **CB-Ox/*n*** and **Thia-Ox/*n***.

Herein, three types of compounds combining the bent 3,5-diphenyl-1,2,4-oxadiazole unit terminally with one or two rod-like mesogenic units through flexible spacers are reported (Scheme 1). The trimesogens **CB-Ox-CB/*n*** combine the 3,5-diphenyl-1,2,4-oxadiazole unit with two nematogenic cyanobiphenyl (CB) units (Scheme 2), in the dimesogens **CB-Ox/*n*** only one CB unit is incorporated, and in compounds **Thia-Ox/*n*** a 3-heptyl-5-phenyl-1,3,4-thiadiazole unit is incorporated (Scheme 3). All compounds form nematic phases over wide temperature ranges, in some cases accompanied by additional nontilted (CybA) or tilted (SmC) mesophases at lower temperatures.

## Results and Discussion

### Synthesis and characterization

The dimesogens and trimesogens were obtained by following the synthetic protocols described in Scheme 2 and Scheme 3 by DCC coupling of the ω-mesogen-functionalized alkanoic acids **2** [66] and **4** [66] with 4-[(4-hydroxyphenyl)-1,2,4-oxadiazol-5-yl]phenyl 4-hexylbenzoate (**3**) [67] and 3,5-bis(4-hydroxyphenyl)-1,2,4-oxadiazole (**1**) [65], respectively. The syntheses of the acids **2** and **4**, and the 1,2,4-oxadiazole substituted phenols **1** and **3** were reported previously in the cited references. The final compounds were purified by crystallization from ethyl acetate/ethanol mixtures. All compounds represent the first examples in their classes and their molecular structure was probed by ^1^H-, ^13^C NMR and microanalytic techniques (see Experimental).

### Investigation of the liquid-crystalline behavior

#### Methods

The obtained di- and trimesogens were investigated by PM (Optiphot 2, Nikon) in conjunction with a heating stage (FP82HT, Mettler) and differential scanning calorimetry (DSC, DSC-7, Perkin-Elmer). The assignment of the mesophases is based on the combined results of optical textures and X-ray diffraction (XRD) studies. XRD investigations on aligned samples were performed by using a 2D wire detector (HI-Star, Siemens AG). Alignment was achieved either in thin capillaries under a magnetic field (*B* = 1 T) or by slow cooling of a small drop of the sample on a glass substrate; the incident X-ray beam was in this case nearly parallel to the glass plate. The transition temperatures, transition enthalpies and observed phase types are collated in Table 1. It is apparent from this table that all of the synthesized di- and trimesogens exhibit liquid-crystalline behavior.

**Table 1:** Phase-transition temperature (*T*/°C) and associated enthalpy values (in square brackets, Δ*H*/kJ mol^−1^) of the synthesized di- and trimesogens.^a^

Compound	Phase transition on heating							Phase transition on cooling						

**CB-Ox-CB/3**	Cr	187 [29.2]	N	320 (dec)	Iso			—^b^						
**CB-Ox-CB/4**	Cr	164 [38.1]	N	246 [4.2]	Iso			Iso	242 [−3.5]	N	123 [−25]	Cr		
**CB-Ox/3**	Cr	162 [40.1]	CybA	210 [<0.01]	N_cybA_	302 (dec)	Iso	—^b^						
**CB-Ox/4**	Cr	130 [36.2]	CybA	148 [<0.01]	N_cybA_	255 [1.7]	Iso	Iso	251 [−1.9]	N_cybA_	146 [<0.01]	CybA	84 [−15.1]	Cr
**Thia-Ox/5**	Cr	144 [41.6]	SmC	173 [0.4]	N_cybC_	224 [2.9]	Iso	Iso	223 [−2.1]	N_cybC_	172 [−0.7]	SmC	114 [−37.2]	Cr
**Thia-Ox/10**	Cr_1_	132 [84.7]	Cr_2_	137 [33.6]	N_cybC_	178 [0.9]	Iso	Iso	174 [−1.0]	N_cybC_	123 [53.4]	Cr		

^a^Peak temperatures in the DSC thermograms obtained during the first heating and cooling cycles at 10 K/min; abbreviations: Cr = crystalline solid; Iso = isotropic liquid, N = nematic LC phase; N_cybA_ = cybotactic nematic phase formed by small SmA-type (nontilted) cybotactic clusters; N_cybC_ = cybotactic nematic phase formed by small SmC-type (tilted) cybotactic clusters; CybA = LC phase formed by extended SmA-type clusters; SmC = smectic C phase; dec = decomposition. ^b^Due to decomposition at the N–Iso transition no cooling curve could be recorded.

#### Trimesogens **CB-Ox-CB/*n***

**Figure 1:**
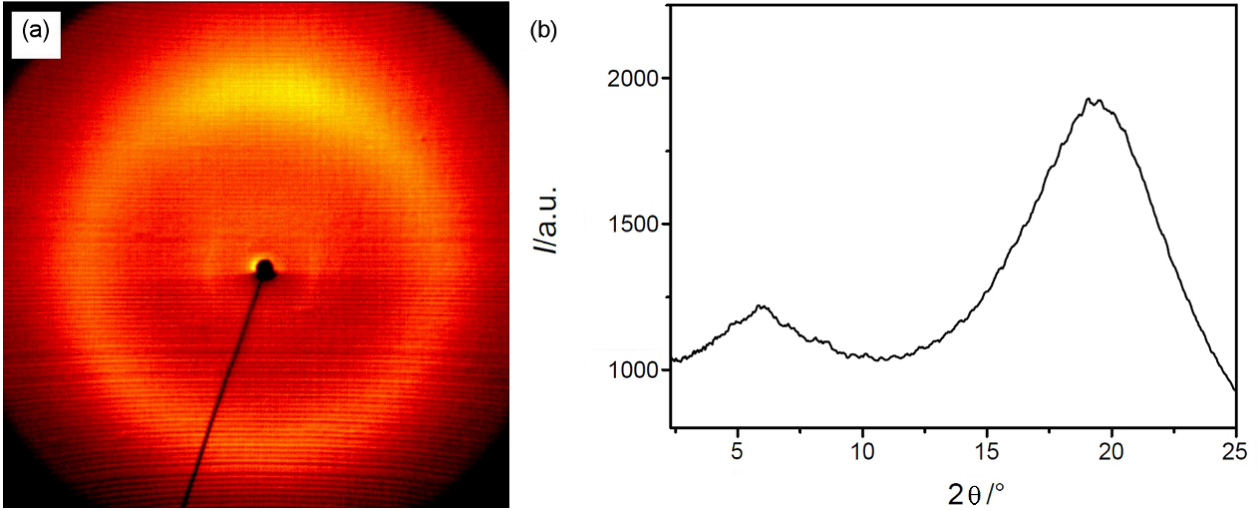
XRD pattern of a (partially) surface-aligned sample of the N phase of compound **CB-Ox-CB/4**: (a) diffraction pattern at 170 °C; (b) θ-scan at 170 °C.

The trimesogens **CB-Ox-CB/*n*** comprising rod-shaped CB segments on either side of the 2,5-diphenyl-1,2,4-oxadiazole unit exhibit exclusively nematic phases (N). Upon cooling of these compounds from the isotropic liquid phase, Schlieren textures were observed, which are evidence of nematic phases [68–69]. These trimesogens have very high clearing temperatures at around *T* = 320 °C for compound **CB-Ox-CB/3** and at *T* = 245 °C for **CB-Ox-CB/4**. The major reason for the much higher nematic phase stability of **CB-Ox-CB/3** compared to the higher homologue may be the result of decreased chain decoupling of the mesogenic units by the shorter spacers and the fact that the spacers are even numbered (considering the ether oxygens and the COO groups as parts of the spacers), which gives a more linear coupling of the mesogenic units. XRD measurement of **CB-Ox-CB/4** confirms the N phase by the presence of two diffuse scattering peaks with maxima at *d*-values of 1.5 nm (very weak) and 0.46 nm, assigned to the average longitudinal distances and the average lateral distances between the molecules, respectively (Figure 1). The longitudinal distance is only about one third of the total molecular length in the most extended conformation, *L*_max_ = 4.6 nm, and hence it is most probably determined by the average length of the three individual aromatic cores. The absence of sufficiently long aliphatic segments and also the electrostatic interaction between the terminal CN groups and the aromatics seem to be responsible for the absence of any smectic phase and the complete mixing of the aromatics in the nematic phase. The low intensity of the diffuse small-angle scattering, due to the low electron-density contrast of these molecules with relatively short spacers does not, in this case, allow further conclusions about the details of the structure of these nematic phases to be drawn.

#### Dimesogens **CB-Ox/*n***

**Figure 2:**
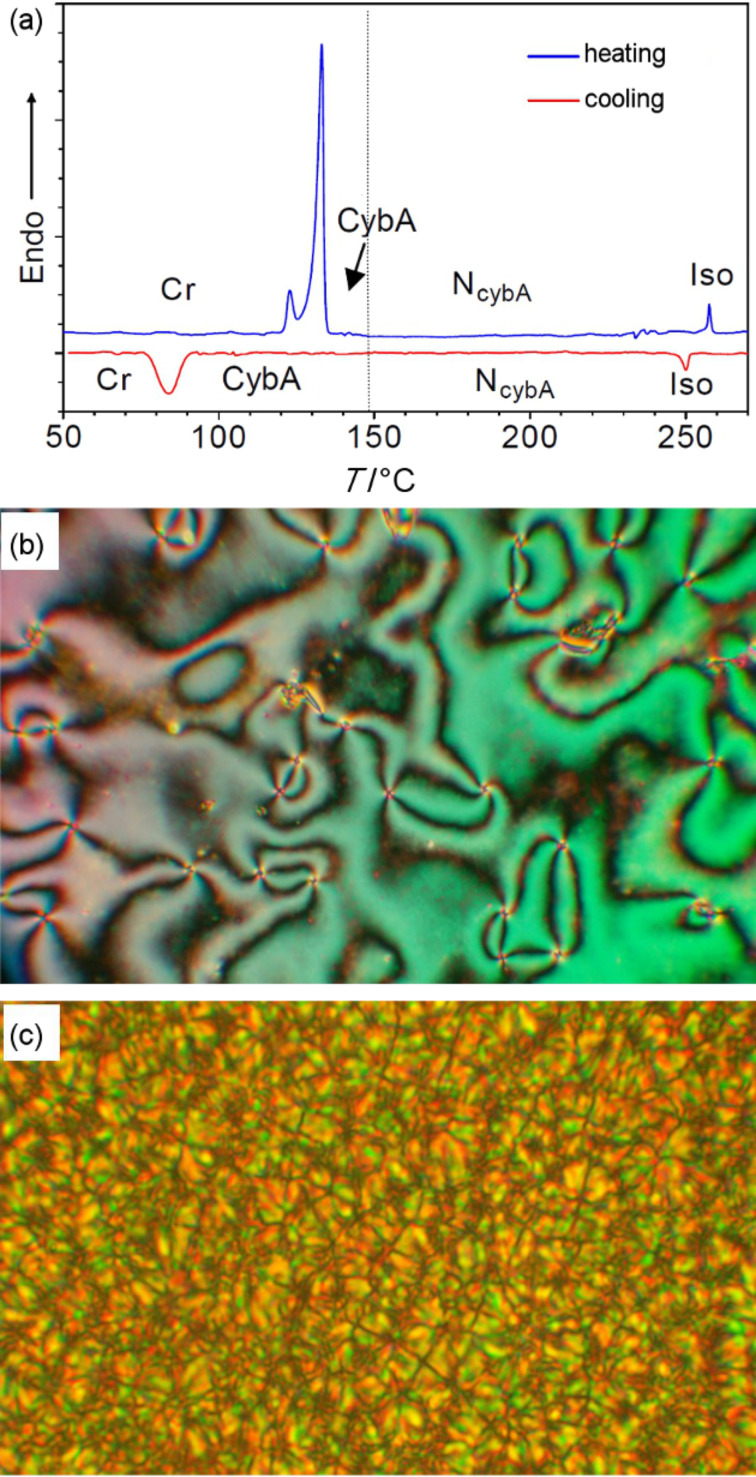
Dimesogen **CB-Ox/4**: (a) DSC traces obtained during initial heating and cooling cycles scanned at a rate of 10 K min^−1^; (b,c) textures as seen between crossed polarizers for a homogenously aligned sample; (b) Schlieren texture of the N_cybA_ phase at *T* = 245 °C; (c) polydomain texture of the CybA phase at *T* = 135 °C.

The dimesogens **CB-Ox/3** and **CB-Ox/4**, having a polar group at one end and an *n*-hexyl tail at the other, exhibit enantiotropic CybA–N phase sequences. Similar to the trimesogens **CB-Ox-CB/*n***, a strong influence of spacer length and spacer parity on the phase-transition temperatures for these dimesogens is also observed. The shorter compound **CB-Ox/3** with even-numbered spacer (O(CH_2_)_3_COO) has much higher temperatures of the CybA–N and N–Iso transitions. Upon cooling a sample of **CB-Ox/4** from the isotropic liquid, the nematic phase forms with a typical highly birefringent Schlieren texture (Figure 2b) [68]. This indicates a predominately homogeneous alignment (director ***n*** on average parallel to the substrate surface) of the sample. On reducing the temperature below 146 °C a change of the texture is observed upon which the Schlieren texture changes into a multidomain texture composed of small fan-like domains (Figure 2c). This texture remains unaltered down to a temperature of 84 °C, below which crystallization takes place. Shearing the sample gives rise to reorganization with homeotropic alignment, which appears completely dark between crossed polarizers, and no birefringence occurs in this homeotropically aligned sample until crystallization sets in. This indicates the formation of a uniaxial mesophase, but surprisingly, no enthalpy change is associated with this transition as observed in the DSC traces (Figure 2a). Similar features can be observed for the shorter homologue **CB-Ox/3** (see Table 1).

**Figure 3:**
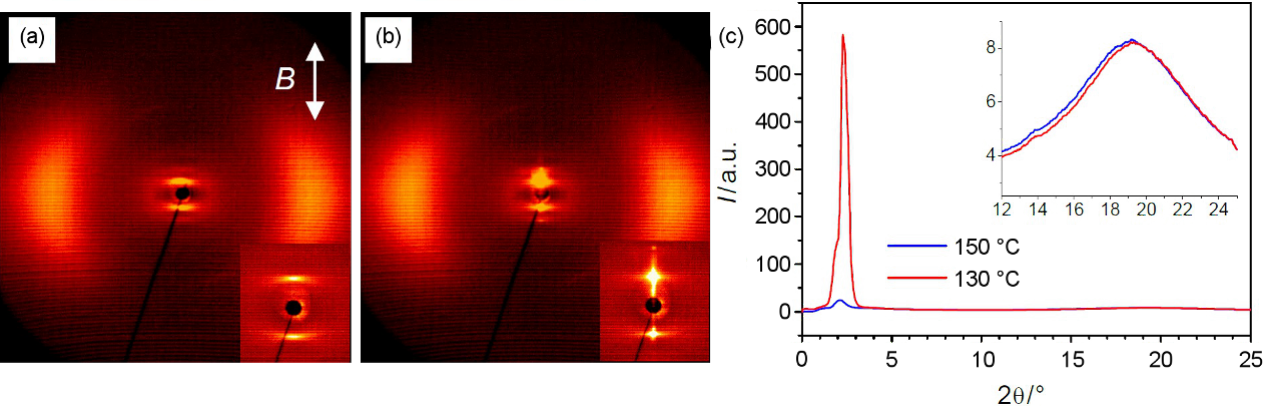
XRD data of the dimesogen **CB-Ox/4**: (a,b) diffraction patterns of a magnetic-field-aligned sample (the direction of the magnetic field is shown as a white arrow), the insets show the scattering in the small-angle region: (a) N_cybA_ at 150 °C; (b) CybA at 130 °C; (c) θ-scans at 150 °C and 130 °C in the small-angle regions of both the N_cybA_ and the CybA phase.

**Figure 4:**
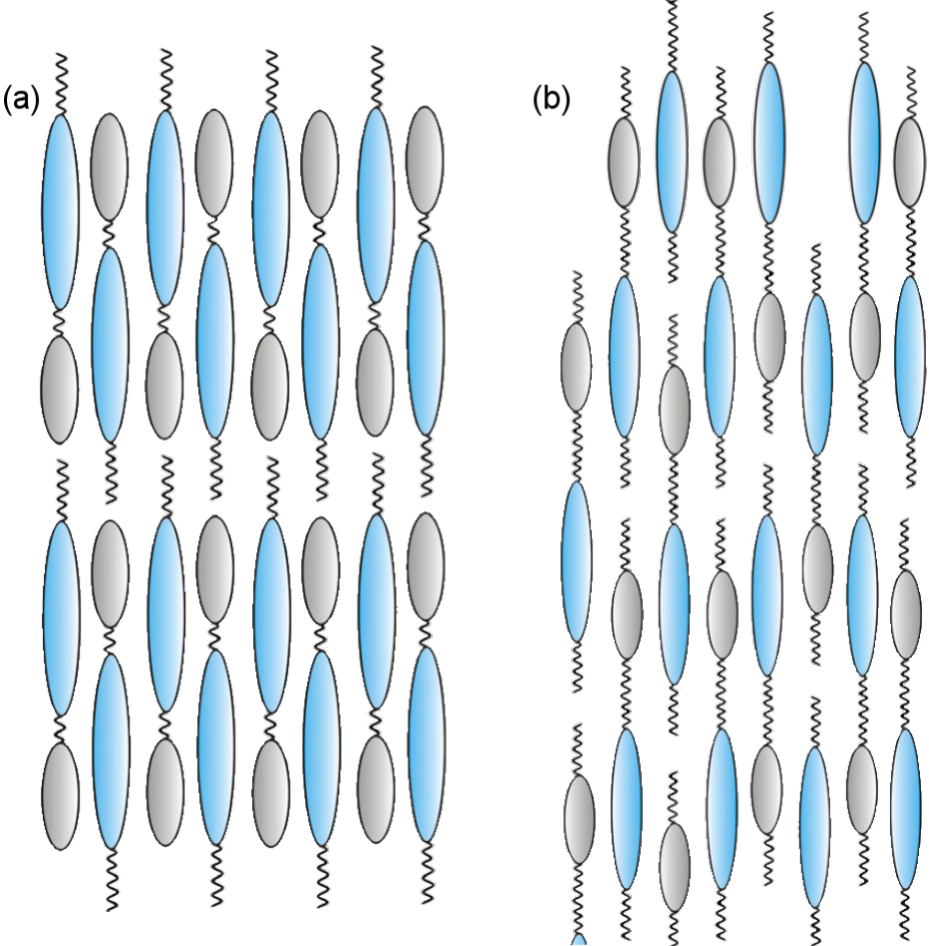
Models showing the suggested organizations of dimesogens in the smectic phases and in the preferred local structure in the cybotactic clusters of the related nematic phases: (a) monolayer structure (*d ~ L*); (b) intercalated structure (*d* ~ ½ *L*). The molecular tilt and the bent-shape of the mesogenic units are not considered.

XRD patterns of the nematic phase of compound **CB-Ox/4**, obtained with a magnetically aligned sample (*B* = 1 T), show diffuse scattering in the wide-angle region with two crescent-like maxima at *d* = 0.46 nm centered on the equator (Figure 3a). In the small-angle region a second diffuse scattering peak with a maximum at *d* = 4.23 nm (*T* = 150 °C) has clear maxima on the meridian of the diffraction pattern. This indicates an arrangement of the molecules with their long axes parallel to the magnetic field direction. The intensity of this small-angle scattering is higher than that of the wide-angle scattering (Figure 3c), which confirms the presence of cybotactic clusters with short-range smectic order [69–74]. Because the maxima of the diffuse peaks in the wide- and small-angle regions are perpendicular to each other the cybotactic clusters are of the nontilted SmA type (N_cybA_) [57,75–76]. The transversal periodicity in these cybotactic clusters (*d* = 4.23 nm) is comparable to the molecular length *L*_mol_ = 4.5–4.6 nm in the most stretched conformation (determined with CPK models). This indicates a kind of monolayer organization of the molecules in the cybotactic clusters. In the SmA-like clusters the alkyl chains are segregated from the aromatic cores, and the aromatics are organized on average antiparallel to each other with complete intercalation of the aromatic units, i.e., the cyanobiphenyls and diphenyl-1,2,4-oxadiazole units appear not to be segregated (Figure 4a). In the aliphatic regions the alkyl chains are interdigitated and conformationally disordered. The size of the SmA type clusters can be estimated from the width of the small-angle scattering at the peak half maximum. The correlation length in the nematic phase at *T* = 150 °C, estimated according to ξ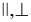 = 2/Δ*q* from the full width at half maximum (Δ*q*) [77], in the longitudinal direction is ξ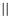 = 5.3 nm and in the transversal direction is ξ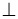 = 1.8 nm. Hence, the dimensions of the cybotactic clusters (*L*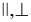) can be approximated to *L*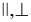 = 3ξ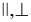 [78], leading to the values *L*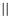 = 16 nm and *L*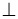 = 5 nm. Accordingly, the clusters are relatively large, composed on average of about 3–4 layers, and about 11 molecules are arranged in the cross section.

On reduction of the temperature further, there is a significant change in the XRD pattern, indicating a phase transition. The diffuse scattering on the meridian becomes sharper and strongly rises in intensity (Figure 3c). In addition, a weak second-order reflection becomes visible (Figure 3b). The maximum of the small-angle scattering is slightly shifted from *d* = 4.23 nm in the N_cybA_ phase at *T* = 150 °C to *d* = 4.31 nm in the low-temperature phase at *T* = 130 °C (Figure 3c). The diffuse outer scattering remains completely diffuse, confirming the absence of any in-plane order. As the maxima of the scattering peaks in the wide- and small-angle regions are perpendicular to each other, the molecules are on average nontilted. Because the position of the small-angle scattering is not significantly shifted, the organization of the molecules should be identical to the model proposed for the SmA-type clusters forming the N_cybA_ phase (Figure 4a). This means that at this phase transition the molecular organization does not change fundamentally, but instead the short-range SmA-like clusters simply become fused to much larger clusters. The correlation length in this phase (*T* = 130 °C) was estimated to 27 nm in the longitudinal direction and 23 nm in the transversal direction. This is somewhat smaller than usually observed for typical SmA phases. Hence, although the correlation length is significantly increased, the smectic order seems not to be truly long-range. It appears that for this reason there is no measurable transition enthalpy for this phase transition (see DSC traces in Figure 2a) as would be expected for a transition to a SmA phase, and hence this phase is tentatively assigned as CybA. Related tilted [69] and nontilted phases [79] with structures intermediate between the N_cyb_ phases and undistorted smectic phases have been observed previously and seem to represent a typical phenomenon for molecules at the borderline between a bent-core and rod-like shape.

#### Dimesogens **Thia-Ox/*n***

The dimesogens **Thia-Ox/5** and **Thia-Ox/10** have a molecular structure that is quite distinct from that of compounds **CB-Ox/*n***. Firstly, they have alkyl tails at both ends, and secondly, they incorporate a 1,3,4-thiadiazole unit, which is not strictly linear (bending angle 162°) [80] and within which the direction of the major dipole moment is perpendicular to the molecular long axis [80–82]. Finally, the spacer units of these thiadiazoles are also significantly longer (*n* = 5, 10) than those used for the related cyanobiphenyl-containing dimesogens (*n* = 3, 4). As a result, LC phases with a tilted organization of the molecules become dominant. The dimesogen **Thia-Ox/5**, with the shorter spacer, displays a SmC–N_cybC_ dimorphism and, remarkably, the SmC phase is removed for the longer homologue **Thia-Ox/10**.

**Figure 5:**
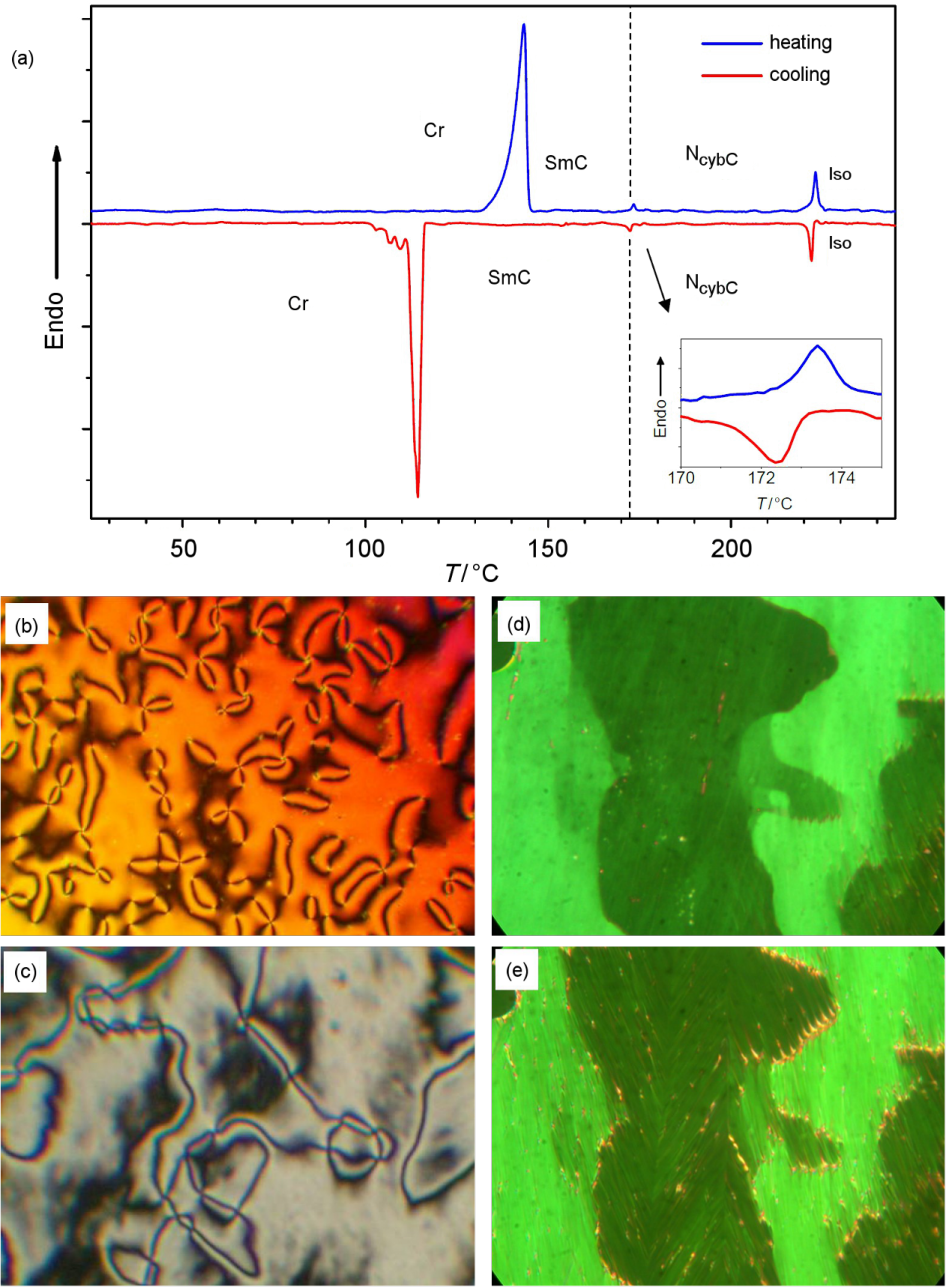
Dimesogen **Thia-Ox/5**: (a) DSC traces obtained during first heating and cooling cycles scanned at a rate of 10 K min^−1^; (b,c) textures as observed between crossed polarizers, (b) N phase having a Schlieren texture at *T* = 220 °C and (c) SmC phase at *T* = 160 °C under homeotropic boundary condition; (d) N phase and (e) SmC phase under homogeneous boundary conditions; the same temperatures as (b,c), observed in polyimide-coated cells, 6 μm.

**Figure 6:**
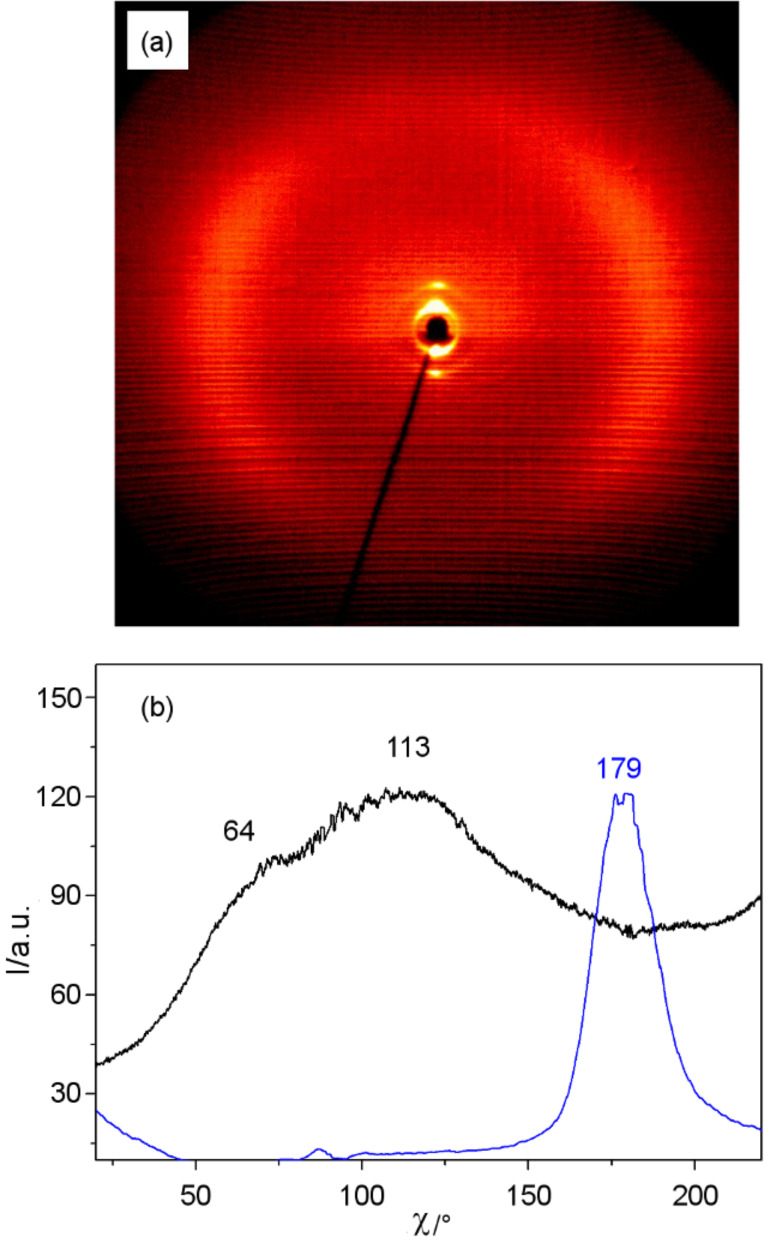
XRD data of the SmC phase of the dimesogen **Thia-Ox/5**: (a) diffraction pattern at *T* = 160 °C; (b) χ-scans over the diffuse scattering in the wide-angle region (black line: 2θ = 15–25°) and the 01 reflection (blue line: 2θ = 1–3°) at 160 °C; the lower part of the diffraction pattern is shaded by the heating stage, therefore, the intensity below the equator is diminished.

A N-to-SmC transition is observed at *T* = 172–173 °C for the dimesogen **Thia-Ox/5** on reduction of the temperature (Figure 5) [68]. In contrast to the N_cybA_–CybA-transition, for which no transition enthalpy could be detected (see Figure 2a), a small but clearly visible enthalpy change is observed for the N_cybC_–SmC transition of **Thia-Ox/5** (Figure 5a). Under homeotropic boundary conditions (Figure 5c) the texture of the low-temperature phase is a Schlieren texture, indicating a biaxial smectic phase. The smectic phase was investigated by XRD of a surface-aligned sample. The 2D XRD profile (Figure 6a) has a sharp Bragg peak (*d* = 4.4 nm) with its second-order reflection located on the meridian, confirming a well-defined layer-like organization. In the wide-angle region there is a diffuse scattering peak with a maximum at *d* = 0.46 nm providing evidence for a liquid-like ordered LC phase. It is split into maxima located beside the meridian, indicating a tilted organization of the molecules in the layers (Figure 6b). From the positions of the diffuse wide-angle scattering peaks a tilt angle of approximately 25° can be estimated. However, it is not possible to decide from the scattering pattern whether there is a synclinic or anticlinic arrangement of molecules in adjacent layers, but the relatively high birefringence of the texture (Figure 5c) is more in line with a synclinic organization. Moreover, as is typical for synclinic tilted SmC phases, domains with different director orientation can be observed in homogeneously aligned samples (Figure 5d and Figure 5e), indicating an optical tilt corresponding to the XRD tilt.

The nearly equal distribution of the diffuse scattering intensity on the left and the right of the XRD pattern (Figure 6a) can be explained with a multidomain structure of the sample with nearly equal contributions of the distinct tilt directions. The layer spacing of *d* = 4.4 nm is smaller than the length of the molecule (*L*_mol_ = 5.3 nm in the most stretched conformation). Considering the tilt, a calculated *d*-value of *d*_cal_ = 4.8 nm would be expected according to *d*_cal_ = *L*_mol_ cos β for the molecule in the most stretched conformation and a reduction to the experimental value of *d* = 4.4 nm is easily possible by conformational disorder. Hence, it is in line with a monolayer structure of the SmC phase in which the aromatic cores and the relatively short spacer units form one type of layer, which is separated by the layers formed by the aliphatic end-chains (Figure 4a). In the layers of the core units, the 3,5-diphenyl-1,2,4-oxadiazole-, 2-phenyl-1,3,4-thiadiazole and the relatively short C_5_ spacer units are mixed, leading to an antiparallel organization of the molecules on average. In the N_cybC_ phases the fundamental structure should be retained but becomes a short-range local structure in the cybotactic clusters.

**Figure 7:**
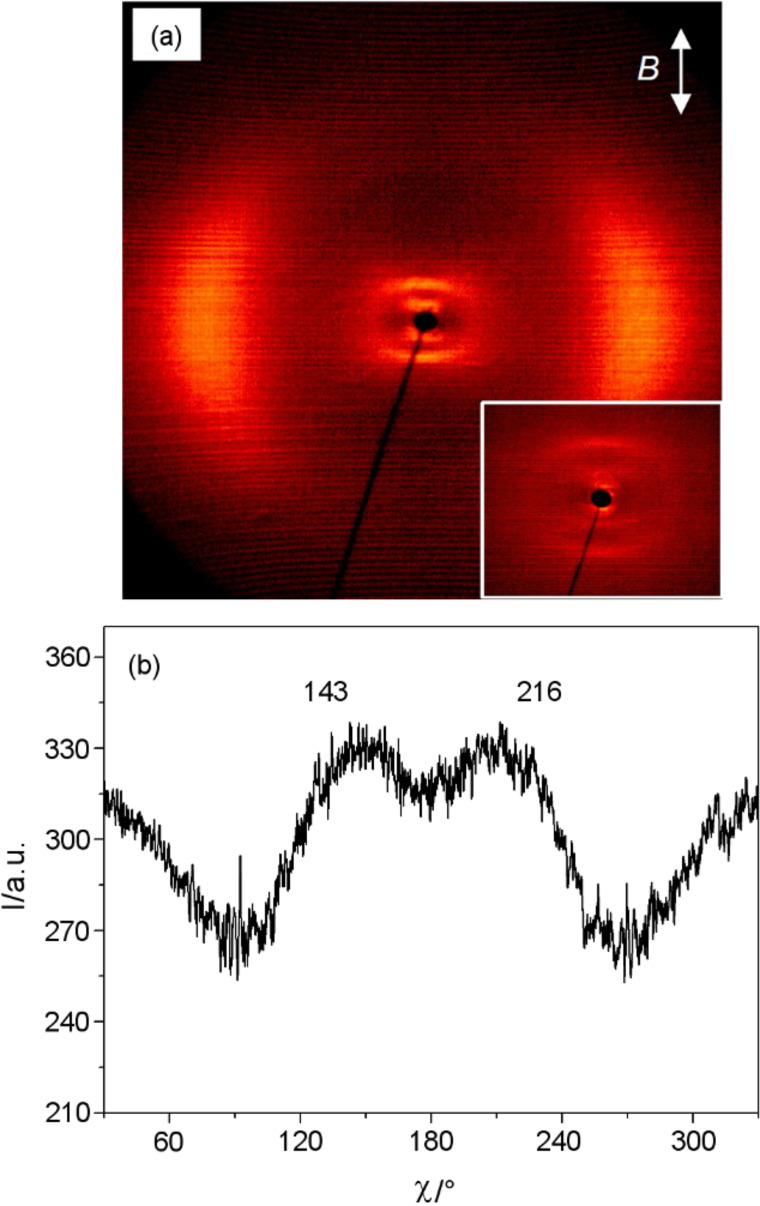
XRD pattern of a magnetic-field-aligned sample of the N_cybC_ phase of the dimesogen **Thia-Ox/10:** (a) diffraction pattern at *T* = 150 °C (inset = small-angle scattering); (b) *χ*-scan over the small-angle scattering (2θ = 2.5–5.0°). Besides the scattering at *d* = 2.5 nm there is a second very weak scattering with a maximum at a smaller θ-angle, corresponding to approximately twice the *d*-value. This would indicate a monolayer structure in the cybotactic clusters, similar to that described for **Thia-Ox/5**. It is unlikely that the much more intense scattering at a higher *d*-value represents the second order of the weak reflection. A possible explanation could therefore be a heterogeneous structure of the sample, probably provided by the interaction of the cybotactic clusters with the surface of the capillary, in which the majority represents the intercalated structure described in the text and the minority is formed by a monolayer structure as found for **Thia-Ox/5**. The proposed formation of a SmC surface layer is basically in line with texture observations made for compound **Thia-Ox/5** (see Figure 8c,d) in which no significant change of the texture is found at the N_cybC_-to-SmC transition. It could be expected that the energetic difference between these two distinct types of layer structures is small, and hence variation of the cluster size under the influence of surface interactions could have an effect on the structure.

Compound **Thia-Ox/10**, with a much longer spacer unit than **Thia-Ox/5**, has exclusively a nematic phase and no transition to a SmC phase could be observed optically on cooling the sample down to 123 °C, at which point crystallization starts. The XRD pattern of a magnetically aligned sample (*B* = 1 T) of this nematic phase shows a crescent-like diffuse wide-angle scattering peak centered on the equator (Figure 7a). In the small-angle region a diffuse scattering peak with a maximum at *d* = 2.5 nm (*T* = 150 °C) is extended to a line parallel to the equator. The χ-scan over this scattering is split into two maxima located beside the meridian (Figure 7b). This indicates the presence of SmC-type cybotactic clusters with a tilt (β) of the molecules of about β = Δχ/2 = 37–39°. The estimated cluster size is *L*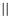 = 5 nm and *L*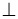 = 1.4 nm, corresponding to about two layers of aromatic cores and about 3 × 3 molecules in the transversal directions. Hence the size of these clusters is significantly smaller than for the N_cybA_ phase of compound **CB-Ox/4**. The *d*-value of the small-angle scattering maximum (*d* = 2.5 nm) is much smaller than the molecular length (*L*_mol_ = 5.9 nm) in the most stretched conformation. Considering the significant tilt the effective molecular length, calculated according to *L*_eff_ = *d*/cos β = 3.2 nm, corresponds to 0.54 *L*_mol_. This small value, close to half the molecular length *L*_mol_, suggests that in the cybotyctic clusters there is a mixed side-by-side packing of the individual aromatic units, separated by the aliphatic domains composed of the mixed terminal chains and C_10_ spacer units (“intercalated” structure, see Figure 4b) [3–4].

Hence, for the dimesogens **Thia-Ox/*n***, elongation of the spacers leads to a change of the molecular organization as indicated by a strong change of the *d*-spacing. Whereas in the case of **Thia-Ox/5** the rigid cores together with the C_5_ spacers are organized side-by-side and separated from the alkyl chains in the monolayer smectic phases and in the cybotactic clusters of the nematic phase (Figure 4a), in the case of compound **Thia-Ox/10** the longer C_10_ spacers cannot be accommodated between the aromatic cores. These long spacers preferably mix with the terminal alkyl chains, and hence only the aromatics are organized side-by-side and the two distinct cores are mixed randomly (Figure 4b). Due to the significant mismatch of the core length of the two mesogenic units, there is some unfavorable overlapping of alkyl chains and aromatics and therefore the layers are not well developed (see Figure 4b). Hence, a long-range smectic ordering cannot develop upon decreasing the temperature, and the molecules only retain an orientationally ordered organization in the SmC-type cybotactic clusters forming the N_cybC_ phase. Hence, for the thiadiazoles **Thia-Ox/*n***, elongation of the alkylene spacer unit connecting the two mesogenic cores removes the SmC phase, which is contrary to the usually observed effect that elongation of alkyl chains stabilizes smectic phases due to improved segregation. The promotion of nematic phases by elongation of an aliphatic spacer unit was previously observed for dimesogens combining two rod-like segments [3–4,83]. As a general rule, for this type of dimesogen the terminal chains must be longer than half the spacer length to form smectic phases [3–4]. According to this rule, the formation of smectic phases should in principle be possible for both compounds, **Thia-Ox/5** and **Thia-Ox/10**. It appears that the very different lengths of the two mesogenic units and probably also the bent shape of these units contribute additionally to destabilization of the smectic phase.

Although these two thiadiazole-containing dimesogens only form tilted LC phases (SmC, N_cybC_), for **Thia-Ox/5** with a relatively short spacer the tilt is significantly reduced (25°) compared to the related 2,5-diphenyl-1,2,4-oxadiazoles without an attached rod-like unit (40–50°) [64–65]. In the dimesogen **Thia-Ox/10**, in which the two mesogenic units are more decoupled, the reduction of the tilt is much less (37–39° tilt). Hence, not only the type of rod-like mesogen, but also the degree of coupling of the rod-like units to the 3,5-diphenyl-1,2,4-oxadiazole core, seems to be important for the degree of tilt.

#### Distinguishing skewed and orthogonal cybotactic nematic phases

**Figure 8:**
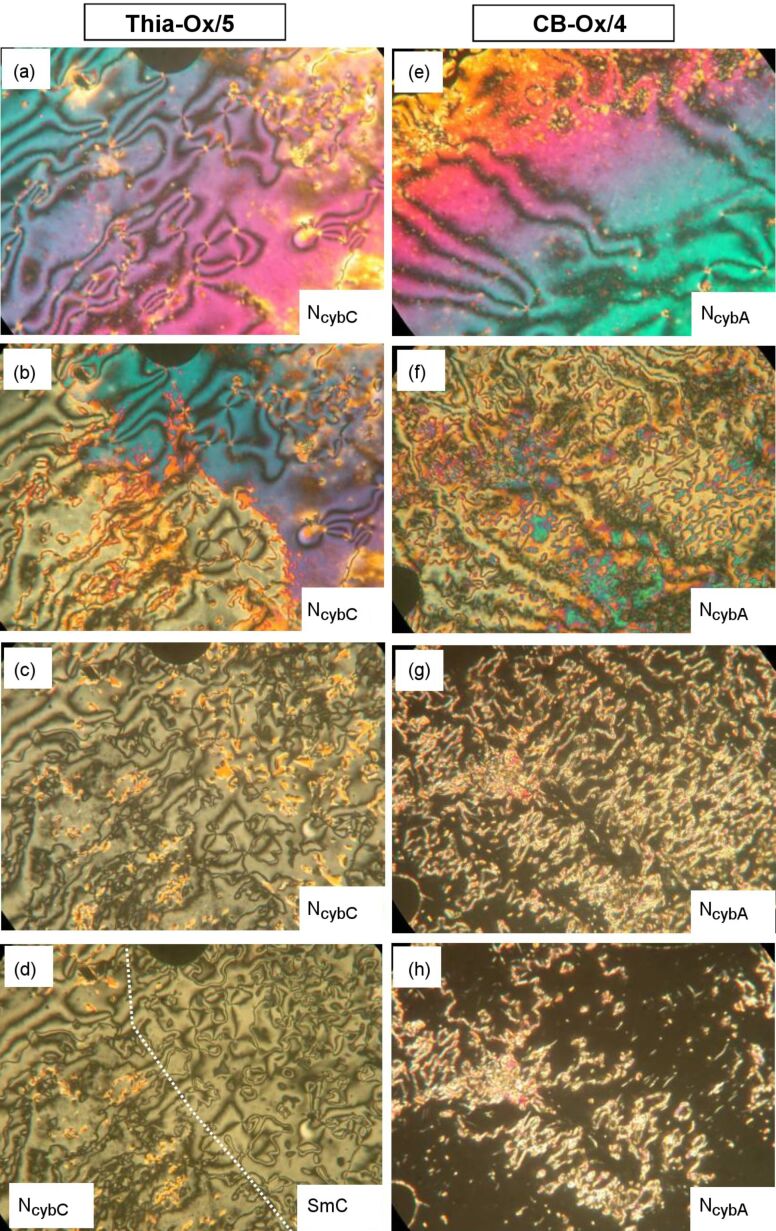
Comparison of the optical textures of distinct types of 1,2,4-oxadiazole based dimesogens as observed between nontreated glass plates (Menzel cover glasses, Menzel GmbH, Braunschweig) between crossed polarizers: (a–d) compound **Thia-Ox/5** at (a) *T* = 206 °C; (b) *T* = 197 °C; (c) *T* = 178 °C; (d) *T* = 173 °C N_cybC_-to-SmC transition, the dotted line indicates the phase boundary; (e–h) compound **CN-Ox/4** at (e) *T* = 254 °C; (f) *T* = 232 °C; (g) *T* = 178 °C; (h) *T* = 150 °C (at *T* = 146 °C in the CybA phase all birefringence has disappeared).

Usually the two distinct types of cybotactic nematic phases, N_cybC_ and N_cybA_, can be distinguished by analyzing the shape of the diffuse small-angle X-ray scattering of magnetically aligned samples, which is split into two maxima beside the meridian for N_cybC_ phases (Figure 8a and Figure 8b) and is nonsplit and centered on the meridian for N_cybA_ phases (Figure 3a). During investigation of compounds **Thia-Ox/5** and **CB-Ox/4** we found that there are also some differences in the optical textures of N_cybC_ and N_cybA_ phases. On cooling from the isotropic liquid state, the textures occur as highly birefringent Schlieren textures (Figure 8a and Figure 8e) in both cases. On further cooling, the texture changes to a much less birefringent appearance for both compounds, attributed to an anchoring transition in which the direction of the molecules changes from parallel to the surface (homogeneous) to perpendicular or tilted with respect to the surface (homeotropic) (Figure 8b and Figure 8f) [52]. This anchoring transition, which depends on the LC material as well as on the properties of the surfaces, could be influenced by temperature-dependent changes of the dielectric anisotropies and steric surface–molecule interactions [52,84], but, as proposed previously, it is most probably also related to the size of the cybotactic clusters forming the nematic phase [69]. At high temperature the clusters are small, and in a homogeneous cell the alignment is determined by the organization of the mesogenic cores parallel to the surface, giving highly birefringent (colorful) textures, as shown in Figure 8a and Figure 8e. As the cluster size grows with decreasing temperature the preferred orientation becomes that with the “layer planes” of the cybotactic smectic clusters being parallel to the cell-surface. In this homeotropic alignment the birefringence is significantly reduced and the textures appear either completely dark (molecules on average perpendicular to the surface, see Figure 8g and Figure 8h) or retain a low birefringence and then appear with gray Schlieren texture (molecules are tilted with respect to the surface, Figure 8c and Figure 8d). Due to surface interactions, the cybotactic clusters can in some cases become aligned at the surfaces even in the nematic phase by forming smectic surface layers, and these layers align the clusters of the bulk sample. In this way the surface anchoring determines the optical properties of the investigated samples. If the surface layer is nontilted (SmA-like) then the sample adopts a homeotropic alignment, which appears completely dark. Indeed for compounds **CB-Ox/*n*** with a N_cybA_–CybA transition the birefringent Schlieren texture disappears upon approaching the transition to the CybA phase (Figure 8g and Figure 8h). In contrast, for compound **Thia-Ox/5**, with a N_cybC_–SmC transition (and also for **Thia-Ox/10** without SmC phase), the birefringent texture, as typical for tilted smectic phases, is retained on cooling down to the transition to the SmC phase (Figure 8c and Figure 8d). In fact the N_cybC_-to-SmC transition is hardly detected by polarizing microscopy (see Figure 8d), but it is clearly confirmed by the DSC peak (Figure 5a) and the XRD pattern (Figure 6a). However, the absence of birefringence in the homeotropically aligned sample does not prove the presence of a N_cybA_ phase, as in a N_cybC_ phase randomization of the tilt direction of the SmC clusters can also result in a completely dark appearance of the homeotropic samples, and in this case birefringence occurs only upon approaching the transition to the SmC phase. Hence, optical investigation can only be used as a first indication of N_cybA_ and N_cybC_ phases if the transition to a homeotropically aligned smectic phase is observed, but it usually requires additional confirmation by XRD of magnetically aligned samples.

## Conclusion

The first examples of end-to-end connected bent-core–rod couples and rod–bent-core–rod trimesogens incorporating a bent 3,5-diphenyl-1,2,4-oxadiazole core have been synthesized and their LC phase behaviors were studied. All molecules show broad regions of nematic phases. The heterotrimesogens with two CB units (**CB-Ox-CB/*n***) form exclusively nematic phases, whereas related dimesogens with only one CB unit (**Ox-CB/*n***) show an additional CybA phase. The nematic phases of these dimesogens represent cybotactic nematic phases [68,71–73] composed of small SmA-like clusters. These cybotactic nematic phases composed of clusters with (on average) nontilted molecules are of significant interest, as restricted rotation around the long axes of the molecules would in this case lead to biaxial nematic phases of the orthorhombic type (N_bo_) [57,73]. In contrast, biaxial nematic phases of the monoclinic type (N_bm_) could be expected for cybotactic nematic phases composed of tilted SmC clusters in which the biaxiality is additionally influenced by the tilt of the molecules. Therefore, the rarely occurring N_cybA_ phases [75–76] are of significant interest in the search for biaxial nematic phases [57]. It is important to note here that the nematic phases of some 3,5-bis(4-hydroxyphenyl)-1,2,4-oxadiazole bis(4-alkyloxybenzoates) have been identified as ferroelectric-like switching cybotactic nematic phases (N_cybCP_) [64–65], but in these N_cybCP_ phases the molecules are strongly tilted (40–50° tilt), such that the field-induced polar and biaxial nematic phases of these compounds represent subtypes of the monoclinic N_bm_ phases. It has been shown here that the combination of the 2,5-diphenyl-1,2,4-oxadiazole core with one CB unit in dimesogens leads to the removal of the tilt and promotes an orthogonal organization of the molecules (CybA, N_cybA_). However, replacing the CB group by a 2-phenyl-1,3,4-thiadiazole core retains the tilted SmC-like organization of the simple 1,2,4-oxadiazole-based bent-core mesogens [81–82].

## Experimental

### Preparation of the trimesogens **CB-Ox-CB/*n***

A mixture of 3,5-bis(4-hydroxyphenyl)-1,2,4-oxadiazole (**1**) [65] (0.39 mmol, 1 equiv), ω-(4’-cyanobiphenyl-4-yloxy)alkanoic acids (**2a**,**b**) [66] (0.78 mmol, 2 equiv) and a catalytic amount of DMAP were dissolved in dry CH_2_Cl_2_ and stirred for 5 min. To the above clear solution, *N*,*N*’-dicyclohexylcarbodiimide (DCC, 1.2 mmol, 3 equiv) was added and stirring was continued for 12 h at rt, followed by solvent evaporation and column chromatography with using EtOAc/*n*-hexane 2:8, to yield a solid, which was further purified by crystallization from EtOAc/EtOH 2:8.

**CB-Ox-CB/3**: 3,5-bis{4-[4-(4’-Cyanobiphenyl-4-yloxy)butanoyloxy]phenyl}-1,2,4-oxa-diazole; colorless solid; yield 71%; ^1^H NMR (400 MHz, CDCl_3_) δ 8.25 (d, *J* = 8.4 Hz, 2H, Ar), 8.16 (d, *J* = 8.0 Hz, 2H, Ar), 7.70 (dd, ^1^*J* = 8.2 Hz, ^2^*J* = 8.4 Hz, 8H, Ar), 7.56 (d, *J* = 8.4 Hz, 4H, Ar), 7.32 (d, *J* = 8.8 Hz, 2H, Ar), 7.27 (d, *J* = 8.0 Hz, 2H, Ar), 7.22 (d, *J* = 8.8 Hz, 4H, Ar), 4.17 (t, *J* = 5.8 Hz, 4H, 2 × OCH_2_), 2.85 (m, 4H, 2× CH_2_COO), 2.32 (m, 4H, 2 × CH_2_); Analysis calcd for C_48_H_36_N_4_O_7_: C, 73.83; H, 4.65; N, 7.18; found: C, 73.76; H, 4.45; N, 6.91.

**CB-Ox-CB/4**: 3,5-bis{4-[5-(4’-Cyanobiphenyl-4-yloxy)pentanoyloxy]phenyl}-1,2,4-oxa-diazole; colorless solid; yield 75%; ^1^H NMR (400 MHz, CDCl_3_) δ 8.22 (d, *J* = 8.0 Hz, 2H, Ar), 8.17 (d, *J* = 8.0 Hz, 2H, Ar), 7.68 (dd, ^1^*J* = 8.4 Hz, ^2^*J* = 8.4 Hz, 8H, Ar), 7.52 (d, *J* = 8.8 Hz, 4H, Ar), 7.28 (d, *J* = 8.8 Hz, 2H, Ar), 7.21 (d, *J* = 8.0 Hz, 2H, Ar), 7.00 (d, *J* = 8.8 Hz, 4H, Ar), 4.09 (t, *J* = 5.2 Hz, 4H, 2 × OCH_2_), 2.71 (m, 4H, 2 × CH_2_COO), 1.97 (m, 8H, 4 × CH_2_); ^13^C NMR (125 MHz, CDCl_3_) δ 175.01, 171.44, 171.21, 168.30, 159.50, 159.48, 154.13, 152.94, 145.19, 145.16, 132.55, 131.57, 131.54, 129.63, 128.84, 128.36, 127.08, 124.48, 122.43, 122.08, 121.76, 119.03, 115.08, 115.06, 110.15, 110.12, 67.46, 67.43, 33.96, 28.52, 21.60, 21.57; Anal. calcd for C_50_H_40_N_4_O_7_: C, 74.24; H, 4.98; N, 6.93; found: C, 73.94; H, 4.74; N, 6.62.

### Preparation of the dimesogens **CB-Ox/*n*** and **Thia-Ox/*n***

A mixture of 4-[3-(4-hydroxyphenyl)-1,2,4-oxadiazol-5-yl]phenyl 4-hexylbenzoate (**3**) [67] (0.34 mmol, 1 equiv), ω-(4’-cyanobiphenyl-4’-yloxy)alkanoic acid (**2a**,**b**) [66] (0.34 mmol, 1 equiv) or ω-[4-(5-heptyl-1,2,4-thiadiazol-2yl)phenoxy]alkanoic acids (**4a**,**b**) [66] (0.34 mmol, 1 equiv) and a catalytic amount of DMAP were dissolved in dry CH_2_Cl_2_. To the above clear solution, *N*,*N*’-dicyclohexylcarbodiimide (DCC, 0.51 mmol, 1.1 equiv) was added and stirred for 12 h at rt, followed by solvent evaporation and column chromatography with EtOAc/*n*-hexane 2:8 to yield a solid, which was further purified by crystallization from EtOAc/EtOH 2:8.

**CB-Ox/3**: 4-(3-{4-[4-(4’-Cyanobiphenyl-4-yl)oxybutanoyloxy]phenyl}-1,2,4-oxadiazol-5-yl)phenyl 4-hexylbenzoate; colorless solid; yield 70%; ^1^H NMR (400 MHz, CDCl_3_) δ 8.24 (d, *J* = 8.8 Hz, 2H, Ar), 8.16 (d, *J* = 8.8 Hz, 2H, Ar), 8.07 (d, *J* = 8.4 Hz, 2H, Ar), 7.64 (dd, ^1^*J* = 8.8 Hz, ^2^*J* = 8.8 Hz, 4H, Ar), 7.49 (d, *J* = 8.4 Hz, 2H, Ar), 7.38 (d, *J* = 8.8 Hz, 2H, Ar), 7.28 (d, *J* = 8.4 Hz, 2H, Ar), 7.21 (d, *J* = 8.8 Hz, 2H, Ar), 6.96 (d, *J* = 8.8 Hz, 2H, Ar), 4.11 (t, *J* = 6.0 Hz, 2H, 1 × OCH_2_), 2.81 (t, *J* = 7.2 Hz, 2H, 1 × CH_2_), 2.67 (t, *J* = 7.6 Hz, 2H, CH_2_COO), 2.23 (m, 2H, CH_2_), 1.63–1.12 (m, 8H, 4× CH_2_), 0.84 (t, *J* = 6.8 Hz, 3H, 1 × CH_3_); ^13^C NMR (125 MHz, CDCl_3_) δ 175.13, 171.24, 168.29, 164.61, 159.36, 154.60, 152.88, 149.89, 145.15, 132.55, 131.74, 130.34, 129.67, 128.87, 128.77, 128.40, 127.11, 126.33, 124.60, 122.69, 122.06, 121.71, 119.03, 115.10, 110.18, 66.71, 36.09, 31.62, 31.04, 30.97, 28.88, 24.52, 22.54, 14.03; Anal. calcd for C_44_H_39_N_3_O_6_: C, 74.88; H, 5.57; N, 5.95; found: C, 74.58; H, 5.27; N, 5.69.

**CB-Ox/4**: 4-(3-{4-[5-(4’-Cyanobiphenyl-4-yloxy)pentanoyloxy]phenyl}-1,2,4-oxadiazol-5-yl)phenyl 4-hexylbenzoate; colorless solid; yield 74%; ^1^H NMR (400 MHz, CDCl_3_) δ 8.23 (d, *J* = 7.2 Hz, 2H, Ar), 8.14 (d, *J* = 6.8 Hz, 2H, Ar), 8.07 (d, *J* = 6.4 Hz, 2H, Ar), 7.63 (dd, ^1^*J* = 6.8 Hz, ^2^*J* = 6.8 Hz, 4H, Ar), 7.48 (d, *J* = 6.8 Hz, 2H, Ar), 7.38 (d, *J* = 6.8 Hz, 2H, Ar), 7.28 (d, *J* = 6.4 Hz, 2H, Ar), 7.20 (d, *J* = 7.2 Hz, 2H, Ar), 6.96 (d, *J* = 6.8 Hz, 2H, Ar), 4.04 (t, *J* = 4.4 Hz, 2H, 1 × OCH_2_), 2.66 (t, *J* = 6.0 Hz, 4H, 2 × CH_2_), 1.93–1.24 (m, 12H, 6× CH_2_), 0.84 (t, *J* = 5.6 Hz, 3H, 1 × CH_3_); Anal. calcd for C_45_H_41_N_3_O_6_: C, 75.09; H, 5.74; N, 5.84; found: C, 74.95; H, 5.61; N, 5.65.

**Thia-Ox/5**: 4-[3-(4-{6-[4-(5-Heptyl-1,3,4-thiadiazol-2-yl)phenoxy]hexanoyloxy}phenyl)-1,2,4-oxadiazol-5-yl]phenyl 4-hexylbenzoate; colorless solid; yield 69%; ^1^H NMR (400 MHz, CDCl_3_) δ 8.24 (d, *J* = 8.8 Hz, 2H, Ar), 8.15 (d, *J* = 8.8 Hz, 2H, Ar), 8.07 (d, *J* = 8.4 Hz, 2H, Ar), 7.80 (d, *J* = 8.8 Hz, 2H, Ar), 7.38 (d, *J* = 8.8 Hz, 2H, Ar), 7.28 (d, *J* = 8.8 Hz, 2H, Ar), 7.19 (d, *J* = 8.4 Hz, 2H, Ar), 6.91 (d, *J* = 8.8 Hz, 2H, Ar), 4.01 (t, *J* = 6.4 Hz, 2H, 1 × OCH_2_), 3.05 (t, *J* = 7.6 Hz, 2H, 1 × CH_2_), 2.66 (t, *J* = 7.2 Hz, 2H, 1 × CH_2_), 2.60 (t, *J* = 7.2 Hz, 2H, CH_2_COO), 1.80–0.82 (m, 30H, 12 × CH_2_, 2 × CH_3_); Anal. calcd for C_48_H_54_N_4_O_6_S: C, 70.74; H, 6.87; N, 5.87; found: C, 70.57; H, 6.54; N, 6.67.

**Thia-Ox/10**: 4-[3-(4-{11-[4-(5-Heptyl-1,3,4-thiadiazol-2-yl)phenoxy]undecanoyloxy}-phenyl)-1,2,4-oxadiazol-5-yl]phenyl 4-hexylbenzoate; colorless solid; yield 72%; ^1^H NMR (400 MHz, CDCl_3_) δ 8.23 (d, *J* = 8.8 Hz, 2H, Ar), 8.14 (d, *J* = 8.8 Hz, 2H, Ar), 8.07 (d, *J* = 8.4 Hz, 2H, Ar), 7.79 (d, *J* = 8.8 Hz, 2H, Ar), 7.37 (d, *J* = 8.8 Hz, 2H, Ar), 7.27 (d, *J* = 8.8 Hz, 2H, Ar), 7.18 (d, *J* = 8.4 Hz, 2H, Ar), 6.89 (d, *J* = 8.8 Hz, 2H, Ar), 3.96 (t, *J* = 6.4 Hz, 2H, 1 × OCH_2_), 3.05 (t, *J* = 7.6 Hz, 2H, 1 × CH_2_), 2.66 (t, *J* = 7.2 Hz, 2H, 1 × CH_2_), 2.54 (t, *J* = 7.2 Hz, 2H, CH_2_COO), 1.77–0.79 (m, 40H, 17 × CH_2_, 2 × CH_3_); ^13^C NMR (125 MHz, CDCl_3_) δ 175.09, 171.85, 169.47, 168.33, 168.11, 164.60, 161.27, 154.57, 153.03, 149.87, 130.34, 129.66, 129.27, 128.83, 128.76, 126.35, 124.43, 122.86, 122.67, 122.11, 121.73, 114.91, 68.18, 36.09, 34.38, 31.62, 31.61, 31.03, 30.17, 30.05, 29.43, 29.31, 29.30, 29.18, 29.12, 29.04, 28.94, 28.87, 28.86, 25.96, 24.84, 22.55, 22.54, 14.03, 14.02; Anal. calcd for C_53_H_64_N_4_O_6_S: C, 71.92; H, 7.29; N, 6.33; found: C, 71.65; H, 7.25; N, 6.29.
